# Non-Invasive Positive Pressure Ventilation Use—Practice Recommendations of the Slovak Society of Pulmonology and Phthisiology

**DOI:** 10.3390/life14030376

**Published:** 2024-03-13

**Authors:** Pavol Pobeha, Imrich Mucska, Robert Vysehradsky, Marta Hajkova, Ivana Paranicova, Pavol Joppa

**Affiliations:** 1 Department of Respiratory Medicine and Tuberculosis, Faculty of Medicine, P. J. Safarik University and L. Pasteur University Hospital in Kosice, 04190 Košice, Slovakia; pavol.pobeha@upjs.sk (P.P.); ivana.paranicova@upjs.sk (I.P.); 2 1st Department of Neurology, Faculty of Medicine, Comenius University and University Hospital in Bratislava, 81102 Bratislava, Slovakia; imucska@gmail.com; 3 Department of Respiratory Medicine and Tuberculosis, Jessenius Faculty of Medicine in Martin, Comenius University in Bratislava, 03601 Martin, Slovakia; robert.vysehradsky@uniba.sk; 4 Department of Respiratory Medicine and Tuberculosis, Faculty of Medicine, Comenius University and University Hospital in Bratislava, 81102 Bratislava, Slovakia; hajkova@unb.sk

**Keywords:** non-invasive positive pressure ventilation, long-term oxygen therapy, respiratory failure, hypercapnia, practice recommendations

## Abstract

Non-invasive positive pressure ventilation (NIPPV) is increasingly used as a treatment method for patients with respiratory failure. The first recommendations for the use of NIPPV in Slovakia were developed by the Slovak Society of Pulmonology and Phthisiology in 2007 and were partially revised in 2015. New scientific evidence prompted the present update, which is based on widely accepted international guidelines and was adapted to address local needs. Important features of the present update include a classification of acute indications for NIPPV into three categories based on the level of supporting evidence, namely 1. definite indications for in-hospital use of NIPPV; 2. possible indications for in-hospital use of NIPPV; and 3. disorders and states in which in-hospital use of NIPPV is not recommended. The current update also reflects the importance of comorbid sleep-related breathing disorders and other chronic respiratory conditions, as well as the use and limitations of continuous positive airway pressure therapy. Since oxygen therapy is often administered along with NIPPV, guidance on the safe use of oxygen in NIPPV-treated patients has also been included. Also, the present update extends the range of its users, addressing the needs of specialists in pediatric respiratory medicine as a novelty.

## 1. Introduction

Non-invasive positive pressure ventilation (NIPPV) has made substantial progress over the past decades. In Slovakia, the first recommendations for NIPPV use were published in 2007 and partially updated in 2015 [1]. In recent years, NIPPV has become very popular among specialists in respiratory medicine, internal medicine, pediatrics, and intensive care in acute settings. Also, the number of patients treated with NIPPV for chronic respiratory failure markedly increased [2]. 

The updated recommendations aim to incorporate new scientific evidence related to NIPPV and reflected by widely accepted national and international guidelines [3,4,5,6,7,8,9,10,11]; adapt to address local gaps in the systematization of NIPPV provision; and to serve as a resource for clinicians, policymakers, and insurance companies to standardize processes aiming to improve the acute and long-term care for patients with respiratory failure in Slovakia. Importantly, the formulation of standard local (Slovakian) recommendations has been requested by policymakers and insurance companies in the country. Such recommendations have both legal and practical consequences; they oblige healthcare providers to ensure minimum recommended procedures, including correct indications, diagnostic equipment, and therapeutic devices in specific healthcare settings. 

## 2. Methodology

The committee for the preparation of the updated practice recommendations included clinicians (pulmonologists and pediatric pulmonologists) and experts in the field of NIPPV and sleep medicine with >10 years (range 11–26 years) of experience in the field of non-invasive ventilation and sleep medicine. The steering committee of the Slovak Society of Pneumology and the writing working group gathered relevant and recent references, analyzed and synthesized the data, and identified limitations of the evidence. The preparation of the recommendations was carried out in the form of in-person and online meetings. 

The practice recommendations in the present update replace the previous ones originally published in 2007 and partially revised in 2015 [1]. The present update was developed under the auspices of the national Slovak Society of Pulmonology and Phthisiology. The preparation included a search of published guidelines from other national and international medical societies, including the joint statement of the European Respiratory Society/American Thoracic Society [3] and the guidelines of the German Society for Pulmonology [4,5], British Thoracic Society [6], Canadian Thoracic Society [7,8], Australian Respiratory Network [9,10], Czech Society of Pulmonology and Phthisiology [11,12], and of relevant related original papers. The content of the present document was adapted to local specific needs and experience of expert NIPPV centers in Slovakia, and it was approved by the Slovak Society of Pulmonology and Phthisiology.

No direct financial support for this document preparation or writing process or any other involvement from the industry was permitted.

## 3. Practice Recommendations for NIPPV Use

***A.***  ***Definition of terms:***

1. Non-invasive positive pressure ventilation (NIPPV) refers to the application of ventilator support in the form of positive airway pressure at two levels (bi-level positive airway pressure [BPAP]) [1], and so-called BPAP devices are used for treatment.
2. Special forms (modes) of NIPPV therapy include assisted pressure support ventilation and volume-assured pressure support ventilation.
3. Nasal, oro-nasal, or full-face masks are used for NIPPV therapy. For previously tracheotomized patients who are unable to tolerate a mask or in whom decannulation is not possible, ventilation using the same BPAP devices can also be provided via the tracheostomy [1,4].
4. In the case of tracheostomy ventilation, it is more appropriate to indicate long-term treatment by home mechanical ventilation. This type of treatment is designated for tracheotomized patients requiring care for tracheostomy. In addition, the devices for home mechanical ventilation are equipped with an internal backup battery [5,6]. Mouthpiece ventilation represents another optional mode included in these devices; it can also be used in ventilator-dependent patients initially ventilated non-invasively as a bridge to tracheostomy ventilation.

***B.***  ***Evaluation of patients for acute in-hospital use of NIPPV:***

1. Arterial blood gases should be assessed in patients with suspected acute respiratory failure in arterialized capillary blood or arterial blood. In chronic respiratory failure, a blood sample is taken during sleep or at rest immediately after waking up.
2. Polysomnography is not generally required to determine the need for NIPPV or to verify treatment effectiveness; it may, however, be considered if there is a clinical suspicion of obstructive or central sleep apnea syndrome.
3. Initiation of NIPPV and verification of treatment effectiveness should be performed during hospitalization.

***C.***  ***Indications for in-hospital use of NIPPV:***

1. Acute respiratory failure or acute deterioration of chronic hypercapnic respiratory failure, including:
  a. Acute exacerbation of chronic obstructive pulmonary disease (COPD) [6,7,9];
  b. Acute cardiogenic pulmonary edema [3,6,7,9];
  c. Acute respiratory insufficiency associated with pneumonia [6];
  d. Acute deterioration of chronic respiratory insufficiency due to neuromuscular diseases (e.g., myasthenia gravis, muscular dystrophy, amyotrophic lateral sclerosis, post-polio syndrome, and high spinal cord lesions), chest wall diseases (e.g., kyphoscoliosis, post-thoracoplasty, and post-tuberculosis sequelae), and obesity hypoventilation syndrome [6,9];
  e. Acute respiratory failure in patients who are immunocompromised (e.g., acquired immunodeficiency syndrome, following organ transplantation, or with hematologic malignancies) [3,9];
  f. The following three conditions must be fulfilled for indicating NIPPV use:
    i. PaCO_2_ ≥ 45 mm Hg;
    ii. Respiratory acidosis with 7.1 < pH < 7.35. Intubation and invasive mechanical ventilation may be considered in patients with severe respiratory acidosis (pH < 7.1) since there is a higher risk of NIPPV failure. NIPPV can be used in patients with severe acidosis when invasive mechanical ventilation is unavailable. While there is no lower limit for the pH value for which an NIPPV trial is inappropriate, patients with low pH require close monitoring due to their high need for invasive mechanical ventilation;
    iii. Respiratory distress, severe breathlessness, tachypnea (≥24 breaths per minute), increased respiration effort with the use of auxiliary respiratory muscles, or thoracoabdominal paradox despite standard medical treatment.
2. Weaning of hypercapnic patients from mechanical ventilation [3,6,7,9];
3. Prevention of respiratory failure following extubation in patients at high risk of hypercapnic respiratory failure [3];
4. Blunt chest trauma (e.g., contusion and serial rib fractures) associated with hypoventilation and respiratory failure [3,6];
5. Conditions following chest surgery, including pulmonary resection or following abdominal surgery, associated with hypoventilation and respiratory failure [3].

***D.***  ***Possible indications for in-hospital use of NIPPV:***

1. No recommendations are available for the appropriateness of NIPPV for the following conditions due to lack of evidence of treatment effectiveness:
  a. Acute exacerbation of bronchial asthma [3,9];
  b. Palliative NIPPV to manage dyspnea in terminal stages of incurable diseases [3].

***E.***  ***Disorders and states in which in-hospital use of NIPPV is not recommended:***

1. Acute respiratory distress syndrome associated with organ failure [13];
2. Acute exacerbation of COPD with hypercapnia and no respiratory acidosis [3];
3. Hypoxemic respiratory failure after extubation following previous invasive mechanical ventilation [3];
4. End-stage cystic fibrosis, except when NIPPV is used as palliative therapy [6].

***F.***  ***Assessment of patients before long-term domiciliary use of NIPPV:***

1. Long-term domiciliary NIPPV therapy indication and prescription fall within the competence of specialists in either respiratory medicine, anesthesiology and intensive care, neurology, or pediatric respiratory medicine. Recommended investigations before the initiation of long-term domiciliary NIPPV include the following [5]:
  a. Medical history, physical examination, and general laboratory investigations;
  b. Blood gases from arterial or arterialized capillary blood. An assessment of arterial blood gases immediately after waking is recommended in suspected cases of sleep hypoventilation;
  c. Pulmonary function testing, including measurements of peak flow and peak-cough flow;
  d. An examination by an ear-nose-and-throat specialist to assess nasal and upper airway patency;
  e. Chest radiography (posteroanterior and lateral projections);
  f. Nocturnal pulse oximetry, limited polygraphy, or polysomnography in suspected cases of sleep-disordered breathing. Capnometry (optional) is highly recommended. Nocturnal pulse oximetry alone is insufficient for diagnosing nocturnal hypoventilation requiring long-term NIPPV;
  g. Echocardiographic examination and electrocardiography in suspected cardiac disorders or pulmonary hypertension.

***G.***  ***Indications for long-term domiciliary use of NIPPV:***

1. Chronic hypercapnic respiratory failure:
  a. Severe, stable COPD if the following criteria are met:
    i. PaCO_2_ > 55 mmHg [5,8];
    ii. PaCO_2_ ≤ 55 mmHg, plus one of the following:
      - At least two hospitalizations for hypercapnic respiratory failure within the past 12 months [11];
      - Progressive increase in arterial carbon dioxide and risk of respiratory acidosis development noted during oxygen testing before initiating long-term domiciliary oxygen therapy (LTOT) [12];
      - Development of symptomatic hypercapnic respiratory failure in patients with COPD and hypoxemic respiratory failure already treated with LTOT [12];
      - Failure of continuous positive airway pressure (CPAP) therapy in patients with comorbid obstructive sleep apnea (residual apnea-hypopnea index > 10 events per hour, persistent oxygen hemoglobin saturation < 90% and/or progressive increase in PaCO_2_) [8,14,15].
  b. Neuromuscular diseases or diseases associated with restrictive ventilatory impairment, including the following:
    i. Neuromuscular diseases (e.g., myasthenia gravis, muscular dystrophy, amyotrophic lateral sclerosis, post-polio syndrome, and other disorders associated with chronic alveolar hypoventilation and respiratory insufficiency) [5,8,10,11,16];
    ii. Chest wall diseases (e.g., kyphoscoliosis, post-thoracoplasty, and post-tuberculosis sequelae) and other disorders associated with severe restrictive ventilatory impairment [5,8,10,11,17];
    iii. High spinal cord lesions associated with restrictive ventilatory impairment, nocturnal hypoventilation, sleep-disordered breathing, or respiratory insufficiency [5,8,10].
  c. Central alveolar hypoventilation [1,5];
  d. Idiopathic alveolar hypoventilation [1];
  e. Obesity hypoventilation syndrome [5,8,10];

Note: For groups (c) to (e), hypoventilation should be confirmed by awake arterial PaCO_2_ levels exceeding 45 mm Hg, supported by either nocturnal capnometry and/or nocturnal desaturation below 90%, alternatively. Unlike all the other groups, hypoventilation in neuromuscular diseases represents an exception, where NIPPV can be indicated with awake arterial PaCO_2_ levels below 45 mmHg if all the following criteria are met: (a) dyspnea at rest; (b) tachypnea with resting respiration rate >24 breaths per minute; and (c) use of auxiliary muscles of respiration at rest.

2. Sleep-disordered breathing if the criteria for CPAP therapy are met [18], plus one of the following:
  a. Failure (lack of effectiveness) of CPAP therapy (i.e., persistent apnea–hypopnea index >10 events per hour or persistent nocturnal desaturations);
  b. CPAP therapy is not tolerated;
  c. CPAP therapy is not advisable, e.g., in patients with severe pulmonary hyperinflation;
  d. Presence of central apneic episodes or periodic breathing on CPAP therapy for obstructive sleep apnea [19];
  e. Another indication for NIPPV use is present.
3. Periodic breathing associated with desaturations below 90% for longer than 5 minutes of sleep time despite oxygen therapy administered with the flow of a minimum of 2 L per minute or periodic breathing persisting for longer than 6–8 weeks while on CPAP therapy effectively eliminating obstructive apneic events [1,18,19].

***H.***  ***Contraindications for the evaluation of long-term domiciliary use of NIPPV*** [6,10]**:**

1. Failure of spontaneous respiration not caused by apneic events;
2. Hemodynamic instability;
3. Acute myocardial infarction;
4. Uncontrolled severe dysrhythmias;
5. Severe gastrointestinal bleeding and /or massive hemoptysis;
6. Massive sputum production;
7. Inability to optimize mask;
8. Patient non-adherence to NIPPV;
9. Non-cooperative patient;
10. Pneumothorax, unless treated by thoracic drainage;
11. Fixed upper airway obstruction;
12. Severe impairment of consciousness (Glasgow coma scale <10), except for hypercapnic encephalopathy or coma;
13. Bulbar and pseudobulbar syndrome with the risk of aspiration (a relative contraindication).

***I.***  ***Verifying the effectiveness of long-term domiciliary NIPPV treatment:***

1. The effectiveness of NIPPV therapy should be verified before its prescription based on one of the following criteria:
  a. Alleviation of hypercapnia:
    i. Reduction in PaCO_2_ by at least 10 mm Hg;
    ii. Reduction in PaCO_2_ by a lesser than 10 mm Hg plus documented significant improvement in PaO_2_ in arterial blood or nocturnal oxygen hemoglobin saturation.
  b. Absence of nocturnal desaturations below 88% with a duration longer than 5 minutes during continual administration of supplemental oxygen with the flow of a minimum of 2 L per minute if LTOT is also indicated [12].

***J.***  ***Indications for supplemental oxygen therapy and NIPPV:***

1. Consider adding oxygen therapy (prescribed as LTOT) to NIPPV if, despite adequate ventilation and normalization of the apnea-hypopnea index, one of the following is present:
  a. Cumulative percentage of total recording time with nocturnal desaturations below 90% during polygraphy, polysomnography, or nocturnal pulse oximetry is >30%;
  b. Nocturnal desaturations below or equal to 88% lasting 5 min or longer recorded by limited polygraphy, polysomnography, or nocturnal pulse oximetry.
2. Another (mandatory) indication for LTOT is present, e.g., comorbid lung parenchymal disorder [19].
3. The oxygen source should be connected to the ventilation circuit via an oxygen T-adaptor between the device and the hose (preferred) or mask. Oxygen flow is titrated to achieve a SaO2 of 90–94%.

***K.***  ***Monitoring the efficacy of long-term home NIPPV:***

1. Periodic assessment (every 6 months) by physicians with training in NIPPV therapy. Shorter assessment intervals may be considered for patients with unstable disease states.
2. Assessments include the following:
  a. Medical history, focusing on sleep quality, morning headaches, daytime sleepiness and fatigue, phlegm production, symptoms of possible respiratory infection, any difficulties associated with the use of NIPPV (e.g., mask seal, facial sores, pressure intolerance, nasal congestion, dry mouth or nose, and gastric distention);
  b. Physical examination, including assessment of the skin in contact with the mask,
  c. Analysis of arterial blood gases and acid-base balance under resting conditions [1,19];
  d. Device check, including filters;
  e. Assessment of treatment compliance (can be checked using telemetry).
3. Therapy settings may be modified (e.g., adjustment of treatment modes or ventilator settings, or re-titration) if complications related to treatment develop or treatment is ineffective. Ineffectiveness of treatment should be considered if any of the following conditions are met:
  a. Average daily use of the device is less than 4 h per day;
  b. Daytime or nocturnal symptoms persist;
  c. Arterial blood gas values are abnormal.

In the case of treatment ineffectiveness, the supervising physician is obliged to inform the patient’s health insurance company about the ascertained facts without delay.

***L.***  ***Patient Education:***

1. Patients should be instructed on the following [5,12,16]:
  a. How to operate the device, including proper cleaning and disinfection methods;
  b. Proper replacement of filters, masks, and tubing;
  c. Troubleshooting. In case of technical problems or need for securing supplies (external circuit elements, filters), the patient or sleep laboratory staff informs the supplier of the device that provides the service and supplies of consumables under the terms of the contract with the patient’s health insurance company.

## 4. Summary

The document “Non-Invasive Positive Pressure Ventilation Use–Practice Recommendations of the Slovak Society of Pulmonology and Phthisiology” reflects the view of experts in non-invasive ventilation in Slovakia and is supported by available and up-to-date widely accepted evidence-based guidelines [3,4,5,6,7,8,9,10,11]. These recommendations have been prepared for physicians and respiratory therapists as an evidence-based clinical practice tool.

Also, the present update extends the range of its users, addressing the needs of specialists in pediatric respiratory medicine as a novelty.

The present update features a new perspective on acute NIPPV use by adopting the distinction of indications from the previous recommendations [1] into groups according to the level of support by currently reviewed guidelines [3,6,7,9]: definite indications, possible indications, and situations in which NIPPV use is not recommended. The main reason was to assist physicians in making decisions in particular clinical situations and to prevent misapplication of non-invasive ventilation. 

It is important to note the novelty of the present recommendations, which adhere to a comprehensive view of chronic respiratory failure and address gaps in the previous regulatory materials implemented in the country. The following four novelty qualities need to be highlighted. First, previous recommendations for NIPPV [1] and LTOT use [20] were issued as two separate documents in Slovakia, thus resulting in the misalignment of oxygen prescriptions in the competence of respiratory physicians. Consequently, the current update of the recommendations overcomes this challenge by specifying the proper use of oxygen therapy for persistent hypoxemia in effectively ventilated patients and also reflects on the importance of co-morbid sleep-related breathing disorders and other chronic respiratory conditions for the consideration of NIPPV use. Second, none of the previous recommendations specified three categories of indication criteria for acute NIPPV use, which led to challenges in the approval (and reimbursement) of NIPPV by the regulatory bodies and insurance companies. The present recommendations rectify this gap. Third, up until now, the recommendations for the use of home mechanical ventilation are non-existent in Slovakia, despite the fact that only such recommendations will enable systematic approval and reimbursement of this therapy in the country. Indeed, the term “Home Mechanical Ventilation” is introduced for the first time in the present NIPPV-dedicated document, specifically constructed for implementation in Slovakia. In the present recommendations, we acknowledge that this type of treatment is designated for patients either with tracheostomy or with a mask in conditions requiring the use of ventilation machines with an internal backup battery, and it allows the combined use of more than one ventilation mode for a single patient (e.g., mask and mouthpiece ventilation) [4]. Fourth, the use of long-term NIPPV for patients with COPD-OSA overlap syndrome is specified in these recommendations, unlike in the previous documents. Finally, the present recommendations expand the list of conditions indicated for NIPPV therapy based on recent evidence-based medicine.

A limitation of the present recommendations is that the GRADE (Grading of Recommendations, Assessment, Development, and Evaluation) methodology [3] was not used. However, most of the reviewed guidelines referenced [3,4,5,6,7,8,9,10,16] to create the present document were based on widely accepted standards for guideline preparation, and consistency across the references supporting each of the recommendations was maintained. 

## 5. Conclusions

In conclusion, the document “Non-Invasive Positive Pressure Ventilation Use–Practice Recommendations of the Slovak Society of Pulmonology and Phthisiology” aimed to provide healthcare professionals in the field of respiratory medicine with both quick and comprehensive practical recommendations for the management of patients with respiratory failure that are not solely restricted to NIPPV.

## Data Availability

Not applicable.
